# Population Pharmacokinetics of Hydroxychloroquine and 3 Metabolites in COVID-19 Patients and Pharmacokinetic/Pharmacodynamic Application

**DOI:** 10.3390/ph15020256

**Published:** 2022-02-21

**Authors:** Jean Claude Alvarez, Benjamin Davido, Pierre Moine, Isabelle Etting, Djillali Annane, Islam Amine Larabi, Nicolas Simon

**Affiliations:** 1 Department of Pharmacology and Toxicology, Paris-Saclay University (Versailles Saint-Quentin-en-Yvelines), Inserm U-1173, FHU Sepsis, Raymond Poincaré hospital, AP-HP, 104 Boulevard Raymond Poincaré, 92380 Garches, France; isabelle.etting@aphp.fr (I.E.); islamamine.larabi@aphp.fr (I.A.L.); 2 Infectious Unit, Paris-Saclay University (Versailles Saint-Quentin-en-Yvelines), Raymond Poincaré Hospital, AP-HP, 104 Boulevard Raymond Poincaré, 92380 Garches, France; benjamin.davido@aphp.fr; 3 Intensive Care Unit, Paris-Saclay University (Versailles Saint-Quentin-en-Yvelines), Inserm U-1173, FHU Sepsis, Raymond Poincaré Hospital, AP-HP, 104 Boulevard Raymond Poincaré, 92380 Garches, France; pierre.moine@aphp.fr (P.M.); djillali.annane@aphp.fr (D.A.); 4 Department of clinical Pharmacology, CAP-TV Aix-Marseille University, APHM, INSERM, IRD, SESSTIM, Hop Sainte Marguerite, 13000 Marseille, France; nicolas.simon@ap-hm.fr

**Keywords:** hydroxychloroquine, desethylhydroxychloroquine, desethylchloroquine, didesethylchloroquine, PK/PD, COVID-19

## Abstract

We develop a population pharmacokinetic model for hydroxychloroquine (HCQ) and three of its metabolites (desethylhydroxychloroquine, Des HCQ; desethylchloroquine, DesCQ; and didesethylchloroquine, didesCQ) in COVID-19 patients in order to determine whether a pharmacokinetic (PK)/pharmacodynamic (PD) relationship was present. The population PK of HCQ was described using non-linear mixed effects modelling. The duration of hospitalization, the number of deaths, and poor clinical outcomes (death, transfer to ICU, or hospitalization ≥ 10 d) were evaluated as PD parameters. From 100 hospitalized patients (age = 60.7 ± 16 y), 333 BHCQ and M were available for analysis. The data for BHCQ were best described by a four-compartment model with a first-order input (KA) and a first-order output. For M, the better model of the data used one compartment for each metabolite with a first-order input from HCQ and a first-order output. The fraction of HCQ converted to the metabolites was 75%. A significant relationship was observed between the duration of hospitalization and BHCQ at 48 h (r^2^ = 0.12; *p* = 0.0052) or 72 h (r^2^ = 0.16; *p* = 0.0012). At 48 h or 72 h, 87% or 91% of patients vs. 63% or 62% had a duration < 25 d with a BHCQ higher or below 200 μg/L, respectively. Clinical outcome was significantly related to BHCQ at 48 h (good outcome 369 +/− 181 μg/L vs. poor 285 +/− 144 μg/L; *p* = 0.0441) but not at 72 h (407 +/− 207 μg/L vs. 311 +/− 174 μg/L; *p* = 0.0502). The number of deaths was not significantly different according to the trough concentration (*p* = 0.972 and 0.836 for 48 h and 72 h, respectively).

## 1. Introduction

**Figure 1:**
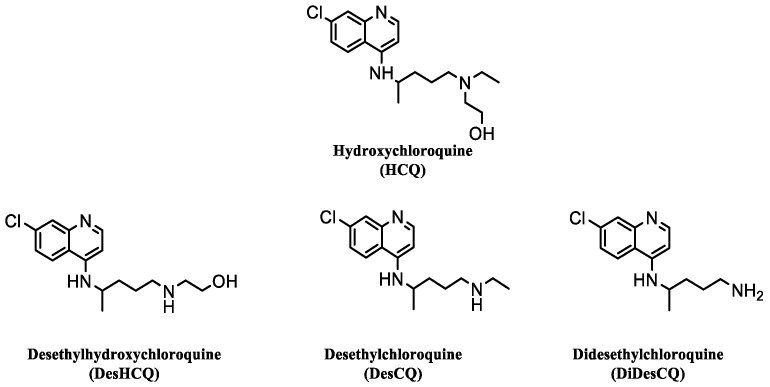
Chemical structures of hydroxychloroquine (HCQ) and its three metabolites.

The pandemic of coronavirus disease 2019 (COVID-19) caused by the severe acute respiratory syndrome coronavirus 2 (SARS-CoV-2) has given rise to the need to identify effective drugs against the virus. There are no specific treatments that have shown sufficient evidence to allow their recommendation, especially in the mild to moderate stages of the disease [1]. Remdesivir, a nucleoside analogue, has shown effect on coronavirus in vitro [2,3]. It is well tolerated and helps to accelerate clinical improvement but has no effect on mortality [1]. Favipiravir appears safe and shows interesting results regarding symptom resolution but does not improve viral clearance. Lopinavir/ritonavir has been associated with an increased risk of gastrointestinal adverse events and it has been shown that probably 50% of patients do not reach enough concentration in plasma and lung with a classical regimen of lopinavir/ritonavir 400/100 mg b.i.d. No significant effects were observed between patients treated with ribavirin or umifenovir and their respective control groups [1]. Hydroxychloroquine (HCQ), an antimalarial drug also used in the treatment of various autoimmune rheumatic diseases including systemic lupus erythematosus (SLE) and rheumatoid arthritis (RA), has been proposed, in association or not with azithromycin (AZT), to treat COVID-19 patients, in some studies with success [4] but some others with failure [5]. Until now, no significant benefits have been clearly highlighted for post-exposure prophylaxis and among hospitalized patients [1], and a large randomized clinical trial has shown that HCQ did not reduce the risk of death among hospitalized patients with COVID-19 disease [6]. However, in this trial, despite using high dosing (patients received a loading dose of 800 mg at baseline and 6 h later, followed by 400 mg 12 h after the first dose and then every 12 h for 9 days), it has been shown in only seven patients that the serum HCQ concentrations were between 350 μg/L and 620 μg/L on day 1 to 4 after initiation [7]. However, 75% of sampling were taken within 4 h of the dose being administered, and were not through concentrations. These concentrations, although obtained in patients hospitalized in medicine, appeared to be lower than those obtained in intensive care unit (ICU) patients treated on a lower dose regimen (200 mg tid) [8]. However, patients who do not receive intensive care can be expected to have better absorption than those hospitalized in the ICU, and therefore higher BHCQ. These results are in agreement with a large variability of the pharmacokinetics parameters (PK) of HCQ in COVID-19 patients, as previously shown for lopinavir [9]. HCQ is almost completely and rapidly absorbed after oral administration, and about 50% of the HCQ in blood is bound to proteins. HCQ is metabolized through hepatic CYP450 isoenzyme 2 D6 to three major oxidative metabolites, desethylhydroxychloroquine (DesHCQ), desethylchloroquine (DesCQ), and didesethylchloroquine (DiDesCQ) [10] (Figure 1).

Only desHCQ is an active metabolite for the treatment of rheumatic disease [11], but nothing is known about the antiviral activities of these three metabolites. Brocks et al. showed that plasma HCQ concentrations were significantly lower and more variable than those in whole blood, suggesting that whole blood would be the optimal matrix to use for the therapeutic drug monitoring (TDM) of HCQ [12]. Moreover, Somer et al. documented a large variability in whole blood HCQ concentrations (∼elevenfold) between patients, confirming a large variability of the PK of HCQ [13]. Several studies have reported a pharmacokinetic/pharmacodynamic (PK/PD) relationship for HCQ (and DesHCQ) in patients treated for SLE and RA [14,15], but few data are available in COVID-19 patients. Thus, the aim of this present study was to evaluate the PK population of HCQ and its three metabolites in COVID-19 patients, and to determine whether a PK/PD relationship could be demonstrated.

## 2. Results

### 2.1. Population

One hundred patients were included, 34 females and 66 males, age = 60.7 ± 15.9 years, body weight = 83.6 ± 20.1 kg, and body mass index (BMI) = 28.9 ± 5.4 kg/m^2^. The demographic and clinical characteristics and clinical outcome of these patients are detailed in Table 1.

#### 2.1.1. Population Pharmacokinetic Analysis

**Figure 2:**
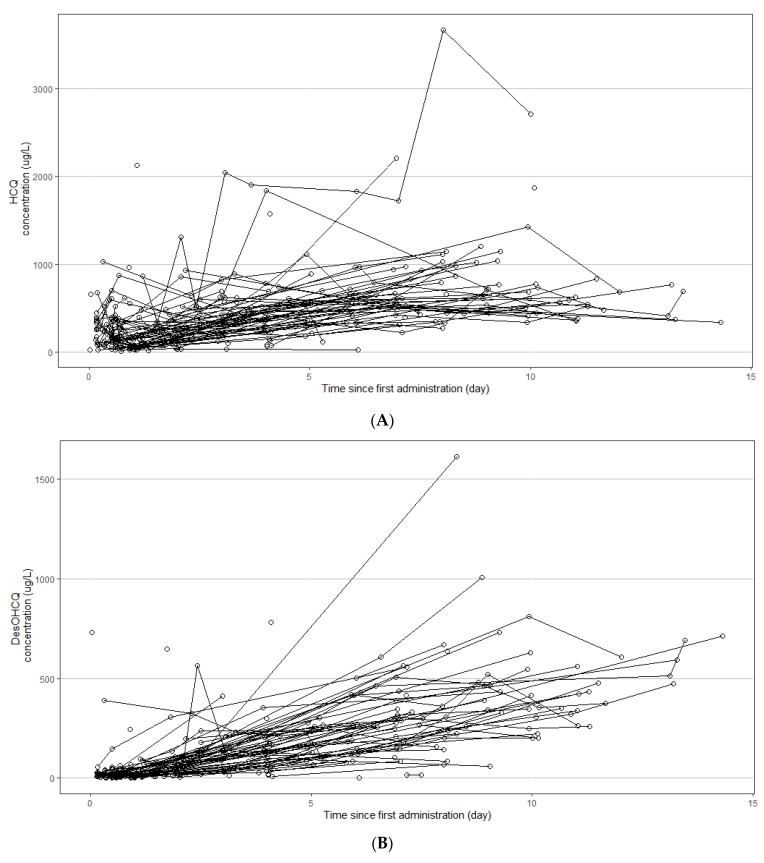

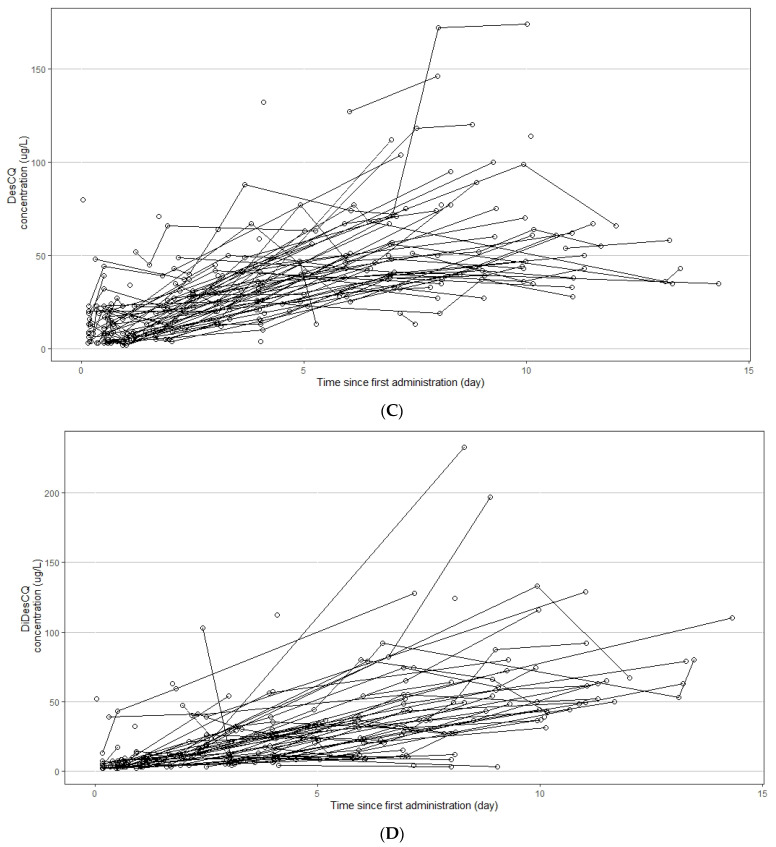
Concentration versus time profiles of hydroxychloroquine (HCQ, **A**), desethylhydroxychloroquine (DesHCQ, **B**), desethylchloroquine (DesCQ, **C**), and didesethylchloroquine (DiDesCQ, **D**) in COVID-19 patients.

A total of 333 blood concentrations were available for HCQ, DesHCQ, DesCQ, and DiDesCQ. Each patient presented between 1 and 9 results. Spaghetti plots of the four compounds are presented in Figure 2A–D, respectively. The best base model for HCQ used four compartments (Figure 2) with a first-order input (KA), a lag time (Lag), a clearance (CL/F), a volume of distribution (VP/F), and clearances to metabolite compartments (DesCQ, DiDesCQ,). The constant rate of absorption KA and the lag time were not estimable appropriately and were fixed at previous published data following a sensitivity analysis [16]. The between subject variability (BSV) was estimated on CL/F and VP/F. The residual unexplained variability (RUV) was modelled as proportional plus additive. Among all the covariates tested on the PK parameters none had a significant effect on the PK parameters. The final PK estimates were well described with small standard errors (Table 2).

Estimates of the shrinkage for CL/F and VP/F were 37% and 4%, respectively.

**Figure 3:**
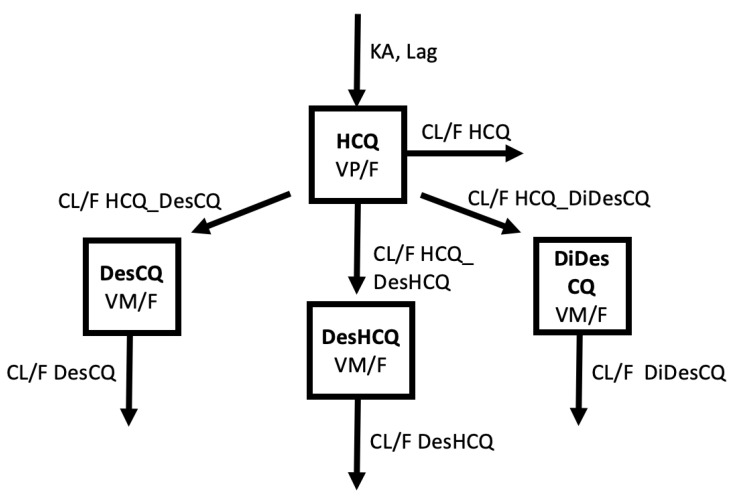
Description of the pharmacokinetic model used to describe HCQ, DesHCQ, DesCQ, and DiDesCQ concentrations.

**Figure 4:**
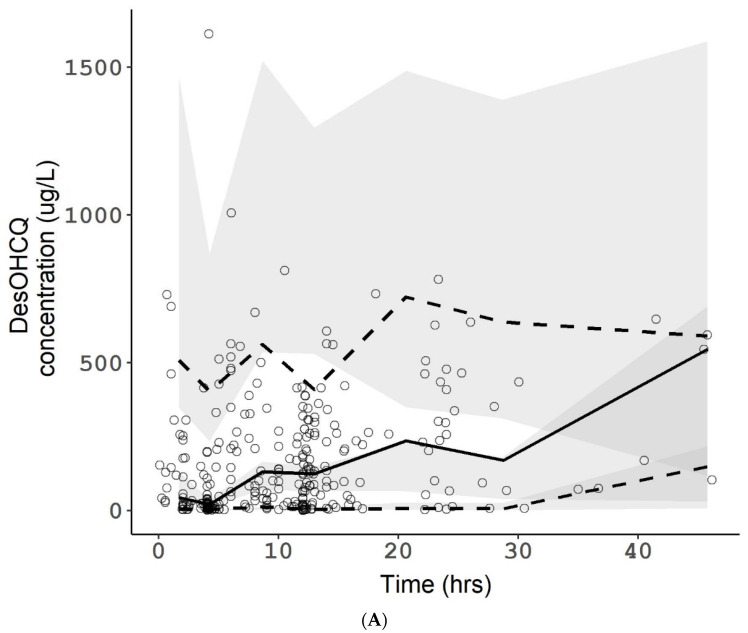

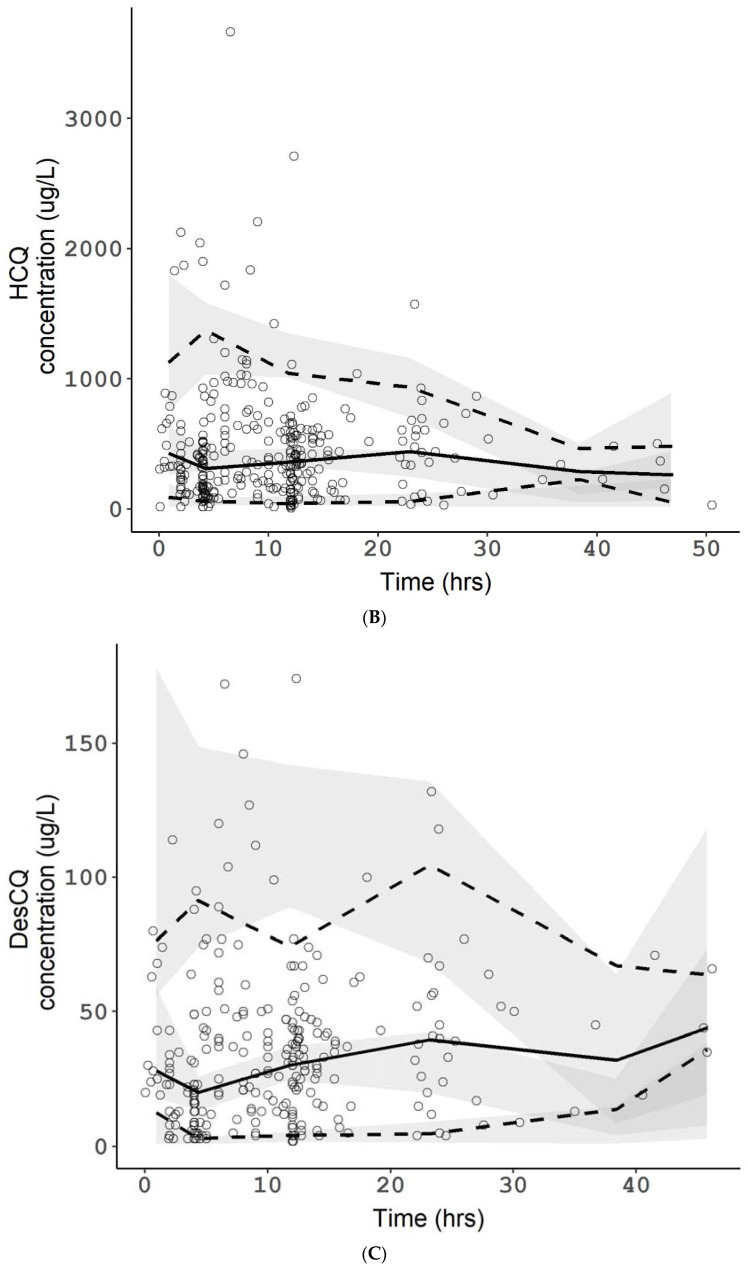

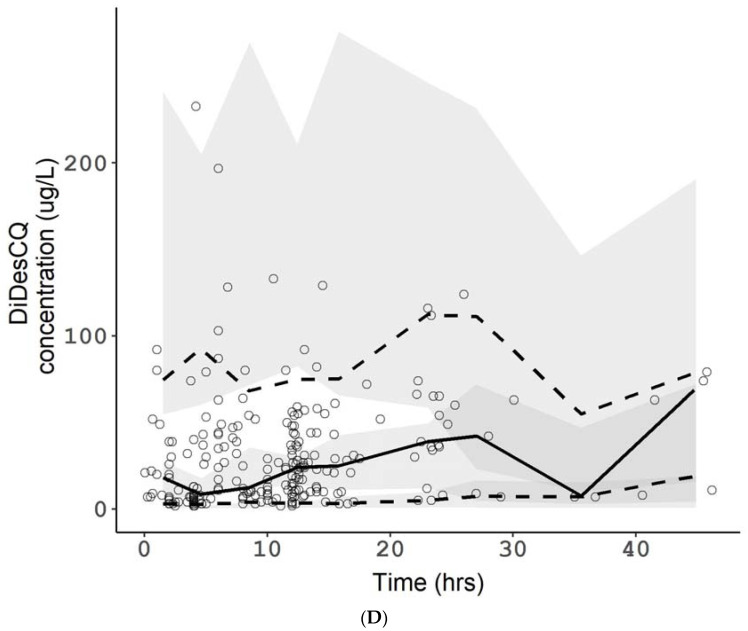
Visual predictive checks of HCQ (**A**), DesHCQ (**B**), DesCQ (**C**), and DiDesCQ (**D**). In each plot, open circles represent observed concentrations. The solid line represents the median of the observed concentrations. The dashed lines represent the 5th and the 95th percentiles of the observed concentrations. The shaded area represents the 90% confidence interval of the 5th, 50th, and 95th percentiles of the simulated concentrations. The horizontal dotted lines represent the lower limit of quantification (LLOQ) of the respective compounds.

For DesCQ, DiDesCQ, and DesOHCQ, the model providing the best description of the data used one compartment for each metabolite with a first-order input from HCQ (modelled as a clearance from HCQ to the metabolite compartment), a first-order output (modelled as a metabolite CL) and a metabolite volume (VM/F) fixed to the volume of the parent HCQ (VP/F) (Figure 3). Other models were tried, such as linking DesOHCQ to DesCQ instead of HCQ, but these models were instable. Goodness-of-plots for the final HCQ, DesHCQ, DesCQ, DiDesCQ, and population pharmacokinetic model were reported in Figure S1 (Supplementary Material). Saturation was also tested with the Michaelis Menten equation from HCQ or from the metabolite compartments but the models’ fits were not improved and the parameters were not appropriately estimated. The BSV of metabolite clearances was estimated and the residual unexplained variability was modelled as proportional plus additive. During the covariate selection process on the metabolite parameters, none significantly decreased the Objective Function. The final PK model with the four compounds (HCQ, DesCQ, DiDesCQ, and DesOHCQ) was used to estimate all PK parameters simultaneously and the results were listed in Table 2. The diagnostic plots as well as the VPC did not show a trend, indicating that the final model adequately describes the PK profile of HCQ and its metabolites (Figure 4A–D).

The fraction of HCQ converted to the metabolites was 75% computed as follows:

FM = Σ(clearances to the metabolite compartments)/(CL/F + Σ(clearances to the metabolite compartments)).

#### 2.1.2. The Pharmacokinetic/Pharmacodynamic Analysis

**Figure 5:**
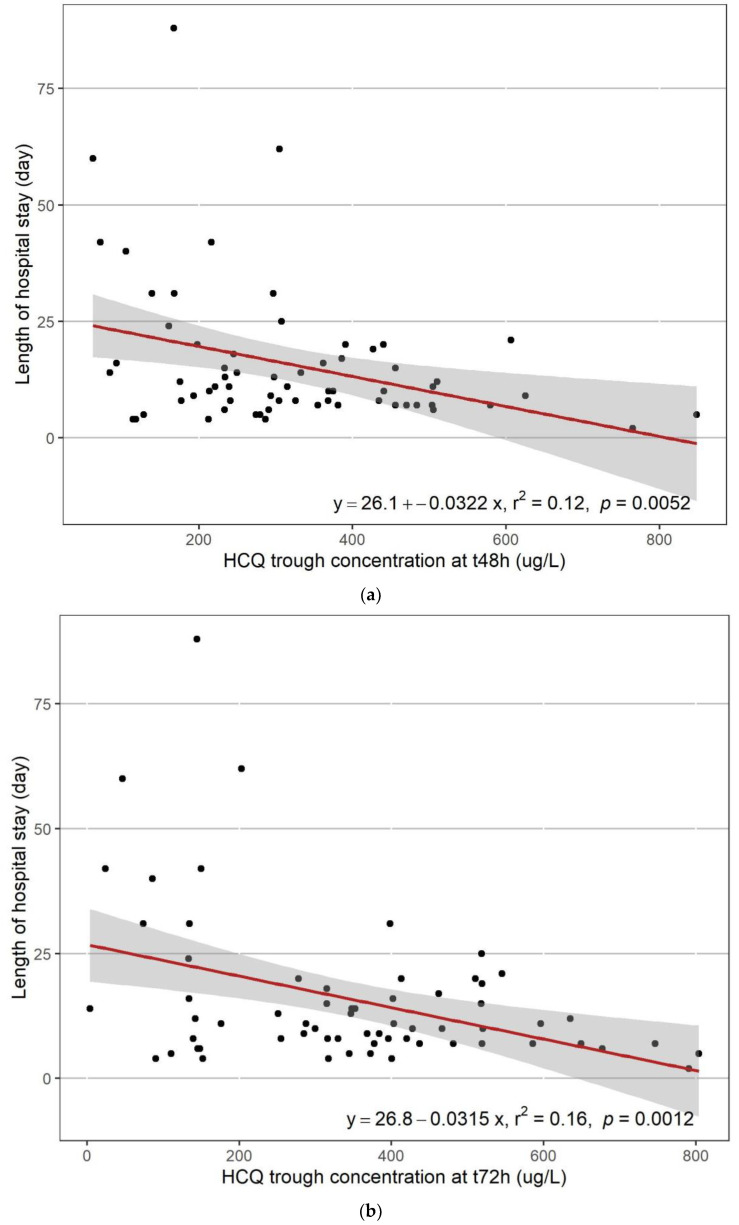
Length of stay versus HCQ blood trough concentrations at t48 h (**A**) and t72 h (**B**).

**Figure 6:**
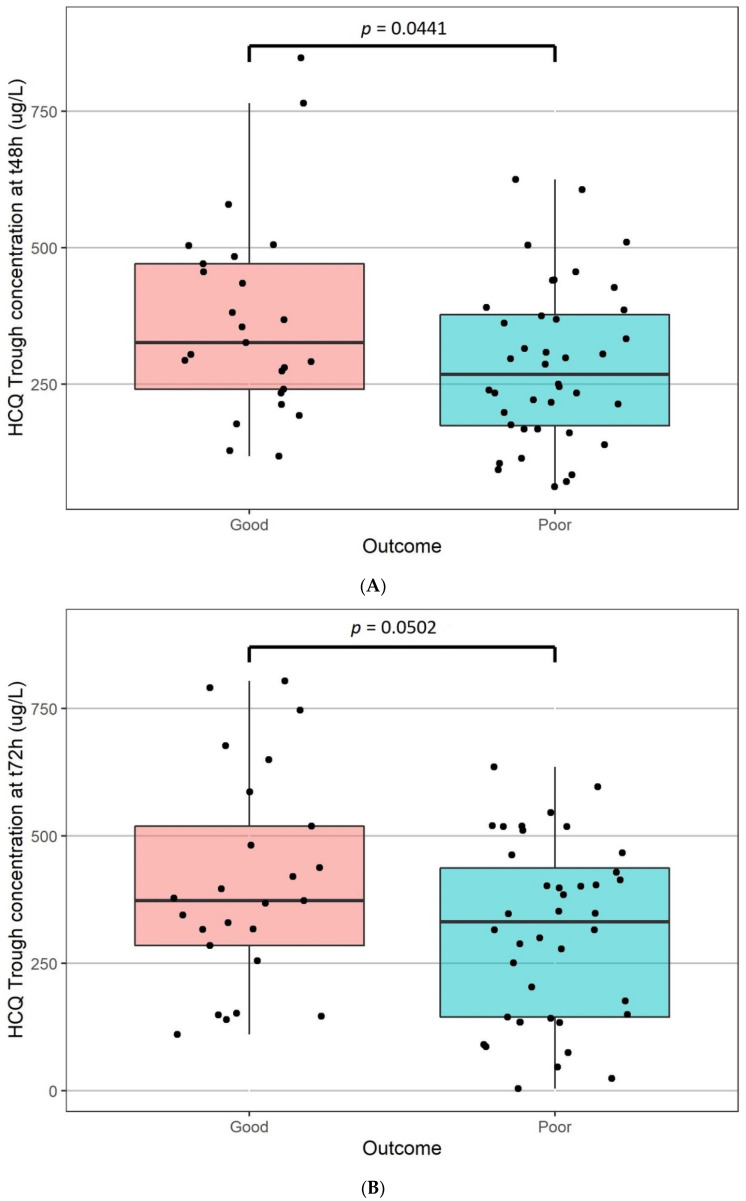
Clinical outcome versus HCQ blood trough concentrations at t48 h (**A**) and t72 h (**B**).

A significant linear relationship was observed between the length of stay and the trough concentrations at 48h (r^2^ = 0.12; *p* = 0.0052) or 72h (r^2^ = 0.16; *p* = 0.0012) (Figure 5A and Figure 5B, respectively). No significant relationship was ound between the length of stay and the HCQ clearance (*p* = 0.836). The clinical outcome was significantly related to trough blood concentrations at 48 h (good outcome 369 +/− 181 μg/L vs. poor outcome 285 +/− 144 μg/L; *p* = 0.0441, Figure 6A) but not at 72 h (good outcome 407 +/− 207 μg/L vs. poor outcome 311 +/− 174 μg/L; *p* = 0.0502, Figure 6B). The number of deaths was not significantly different according to the trough concentration (*p* = 0.972 and 0.836 for 48 h and 72 h, respectively). No significant relationship was found after Holmes correction between the length of stay, the clinical outcome, and the number of deaths versus the trough concentrations at 48 h and 72 h of the three metabolites.

**Figure 7:**
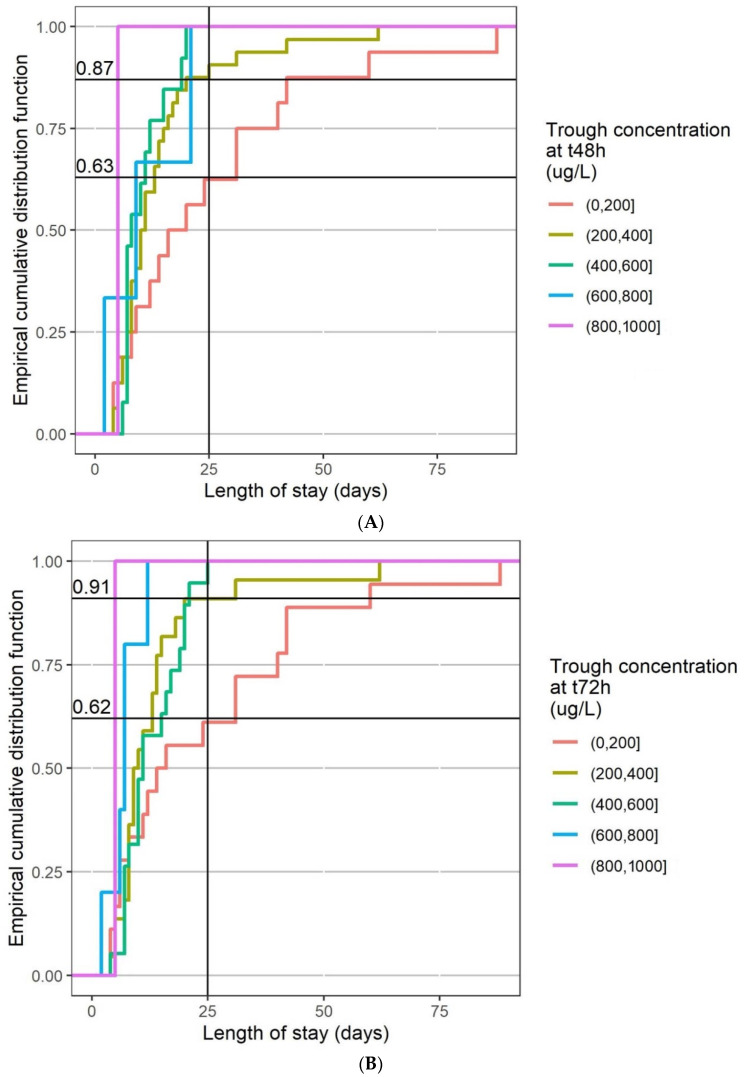
Empirical cumulative distribution function of length of stay by range of HCQ blood trough concentrations at t48 h (**A**) and t72 h (**B**).

The empirical cumulative distribution function of the length of stay was plotted for different HCQ trough concentration ranges: 0–200 μg/L, 200–400 μg/L, 400–600 μg/L, 600–800 μg/L, and above. Figure 7A,B depicts the results at 48 h and 72 h, respectively. More than 85% of the patients with a trough blood concentration higher than 200 μg/L at 48 h had a length of stay below 25 days. They were less than 65% having a length of stay below 25 days among those with a trough blood concentration lower than 200 μg/L at 48 h. At 72 h, 91% of patients versus 62% had a length of stay below 25 days with a trough blood concentration higher than 200 μg/L.

**Figure 8:**
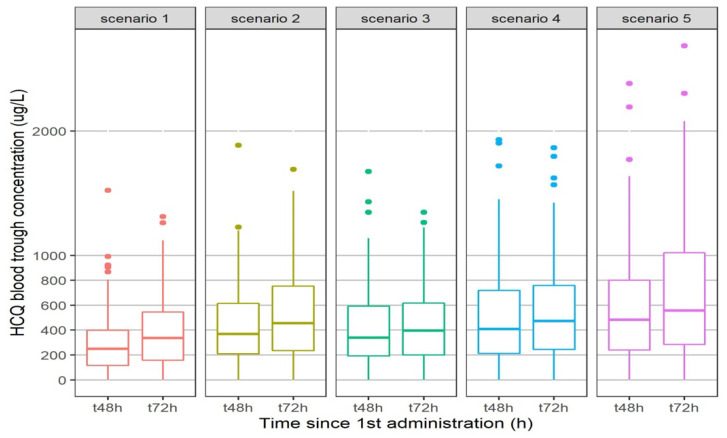
Simulation of HCQ trough concentrations at t48 h and t72 h according to different regimen. Scenario 1:200 mg bid; Scenario 2:200 mg tid; Scenario 3:400 mg bid on Day 1 followed by 200 mg bid; Scenario 4:400 mg bid on Day 1 followed by 200 mg tid; Scenario 5:400 mg bid.

Figure 8 describes the trough blood concentrations of HCQ for five regimens. All regimens except one (200 mg bid) had less than 25% of patients with a concentration below 200 μg/L at 48 h or 72 h. Either 200 mg tid or a 400 mg bid loading dose at day 1 are required to reach a concentration higher than 200 μg/L at 48 h.

## 3. Discussion

In this large clinical study, we were able to model the population PK of HCQ and its three metabolites in 100 COVID-19 treated patients using 333 BHCQ and its three metabolites from these patients. We identified a significant linear relationship between the length of stay in hospital and clinical outcome and the trough BHCQ at 48 h, without a relationship with mortality.

A population PK model for HCQ and its three metabolites used in COVID-19 patients was developed giving an adequate description of the PK of HCQ. In our model, the fraction of HCQ converted to the metabolites was 75%, in agreement with the 16% of an oral dose excreted in the daily urine as unchanged drug found in previous study [17].

We decided to measure BHCQ instead of plasma or serum concentration because plasma HCQ concentrations has been shown to be significantly lower and more variable than those in whole blood [12]. In fact, HCQ accumulates in red blood cells and granulocytes as chloroquine, with a volume of distribution much higher in plasma (around 40,000 L) than in blood (around 5000 L) at steady state [18]. In our model, the value of the volume of distribution of HCQ in blood found was also very high at 1850 L. This high volume of distribution explains why Ruiz et al. found a high ratio of epithelial lining fluid/plasma concentrations (around 40 with a 400 mg x 1 dosage regimen) [19]. Moreover, it also explained the very high terminal half-life of HCQ, more than 40 days, leading to a steady-state concentration achieved in more than 6 months. For this reason, it is important to have early biomarkers that predict good clinical outcomes in order to modify the dosage regimen rapidly. We found here that BHCQ at 48 h could be predictive of the clinical outcome of the patients. A concentration above 200 μg/L 48 h after initiation seems to be associated with a better outcome than a concentration lower than 200 μg/L. According to our model, a regimen of 200 mg tid or rather a loading dose of 400 mg bid on the first day followed by a regimen of 200 mg bid or tid is required in order to achieve this threshold of 200 μg/L at 48 h. With a regimen of 400 mg bid, some patients could have a BHCQ higher than 2000 μg/L (Figure 8), with a risk of cardiotoxicity [20,21].

Many studies have been conducted using HCQ in COVID-19 patients, associated or not with AZT, with different results. Three uncontrolled studies showed that HCQ treatment was significantly associated with viral load reduction or disappearance, with the effect reinforced by AZT [4,22,23]. In one of these studies, mean serum HCQ concentration was “evaluated” after the addition of BHCQ and one metabolite whose concentration was deduced from UV absorption (although the method used was not very clear). This mean serum HCQ concentration was 460 ± 200 µg/mL (*n* = 20 out of the 26 included patients), but no details were given on the days of sampling after the initiation of treatment, which seems different according to the patient [4]. In another study [23], the length of stay in hospital was found shorter in HCQ treated patients (8.6 days ± 5.2 days) vs. control (standard care, 12.1 ± 9.6 days) and even shorter if AZT was added to HCQ (7.1 ± 3.2 days, *p* = 0.04). These publications are conflictual because of the exclusion of some patients which may have biased the results [24]. On the other hand, some other studies did not show any effect of HCQ ± AZT in COVID-19 patients, either in hospitalized patients [5,25] or in outpatients [26,27]. However, to our knowledge, in all these studies, BHCQ was never measured, which could impact the clinical results. For the first time, we found that the length of stay in hospital could be correlated with BHCQ, showing the interest of therapeutic drug monitoring of BHCQ.

BHCQ between 500 and 2000 μg/L has been suggested as a therapeutic range [28] at least in SLE. The mean steady-state whole blood HCQ, DesHCQ, and DesCQ concentrations from 37 SLE patients treated with HCQ (daily dosing range 200–600 mg/day) were 1336 ± 621 μg/L, 1037 ± 721 μg/L, and 115 ± 54 μg/L, respectively [29]. Our results appear to be of the same order but lower since our patients were not at steady-state, with BHCQ around 500–1000 µg/mL, DesHCQ around 500 µg/mL, DesCQ and DiDesHCQ around 50–100 µg/mL. No therapeutic concentration range has been defined in COVID-19 patients. According to the heterogeneity of the reported EC_50_ value, ranging from 241 to 1400 μg/L for HCQ at 48 h and 72 h post-infection, respectively [30,31], and even 5800 μg/L in one study [32], it appeared difficult to determine such a therapeutic concentration range, especially since the concentration in the tissues and in particular the lungs seem much higher [19]. According to our results, a BHCQ higher than 200 μg/L at 48 h after the beginning of treatment seems necessary (Figure 7A) and predictive of better outcomes during hospitalization than patients with HCQ BHCQ lower than 200 μg/L. These results showed the interest of TDM for this drug, in accordance with Tecen-Yucel et al.’s letter, in which they suggest that individual dose modification by TDM could help to achieve optimal outcomes [33]. However, no relationship was observed with mortality, probably in accordance with the fact that HCQ could act on the early stage of the illness but not when the disease is severe [22]. However, 25% of our patients were considered as severe since they were hospitalized in ICU.

Pharmacodynamic data were missing for 35 patients. However, we decided to include all the 100 patients for the PK analysis. These 35 missing patients had only one sampling of blood HCQ, and we did not have access to their clinical outcome and their length of stay in hospital. We only knew if they were dead or alive upon leaving hospital.

## 4. Materials and Methods

### 4.1. Population

All procedures performed in this study involving human participants were in accordance with the ethical standards of the institutional and/or national research committee and with the 1964 Helsinki Declaration and its later amendments or comparable ethical standards. All patients in ICU were included in the RHU (Hospital–University Research in Health) RECORDS program on sepsis (from ANR, French National Agency of Research, ethics committee Est 1, Dijon, No. 20.00479.051415), and gave their informed consent to participate to this program. Patients in medicine wards were included in the study registered on ClinicalTrials.gov (accessed on 18 February 2022, NCT04453501, ethics committee CESREES/Health Data Hub, No. MR1811190620). All adults admitted to the intensive care unit (ICU) or in the medicine wards for a COVID-19 infection confirmed by severe acute respiratory syndrome coronavirus-2 (SARS-CoV-2) reverse transcriptase-polymerase chain reaction (RT-PCR) and/or a compatible pulmonary computerized tomography-scan and treated with HCQ (Plaquenil 200 mg^®^) with at least one sampling available for measurement of blood concentrations were retrospectively included in this study. Patients received treatment as recommended by Yao et al.’s study [30]) and the French Society of Pharmacology and Therapeutics [20]: 200 mg bid or tid, possibly preceded by a loading dose of 400 mg bid the first day. Some patients simultaneously had AZT treatment at a dose of 250 mg bid on the first day, followed by 250 qd.

### 4.2. HCQ and the 3 Metabolites Measurement

A therapeutic drug monitoring of HCQ was recommended in order to not exceed a cardiac toxic blood concentration of 2000 μg/L [20,21]. Sampling of blood for measurement of the 4 compounds was carried out every two days when possible. Blood was collected in a sample tube containing heparinate Li as anticoagulant. Tubes were immediately brought to the laboratory. Blood HCQ and metabolites were quantified using a validated and accredited (COFRAC, Comité Français d’Accréditation) turbulent-flow liquid chromatography mass spectrometry method. Briefly, after the addition of internal standard (HCQ-D_4_) and 100 µL of NaOH 1 M, 200 µL of sample, calibrator, or quality control were extracted by a diethyl ether/chloride methylene (3/1, *v*/*v*) mixture. After centrifugation, supernatant was evaporated to dryness. The residue was reconstituted in 150 µL of mobile phase and 10 µL were injected onto the chromatographic system, consisting of a Turboflow^®^ on-line extraction system used on mode LX, a CTC auto sampler and a triple quadruple mass spectrometer TSQ QUANTUM Access MAX equipped with an electrospray ionization interface. The analytical HPLC column was a Hypurity C18 (150 × 2.1 mm × 35 µm) column. Data analysis wasperformed using an LCQuan™ 2.7 software package (all ThermoFischer Scientific^®^, Les Ulis, France).

### 4.3. Pharmacokinetic Analysis

A population pharmacokinetic analysis was performed using non-linear mixed effect modelling as implemented in NONMEM version 7.4.1 [34]. Data management, statistical analysis, and graphical outputs were realized with R version 4.0.3 [35] and the ggplot2 package [36]. Wings for NONMEM version 743 was used as a “front end” for the NONMEM program [37]. The first-order conditional estimate method with the interaction option (FOCE-I) was used to fit the models. Compartmental pharmacokinetic models were coded using ADVAN 5 and TRANS 1 subroutines.

Model selection started with only HCQ concentrations and once defined each metabolite (DesHCQ, DesCQ and DiDesCQ) were added one by one to the dataset to define the relative metabolite pharmacokinetic parameters. All concentrations were expressed as µmol/L to allow for incorporation in the model. The conversion to μg/L unit was performed for the graphics. During each step a base model was first defined. This included the use of one-, two-, or three-compartment models with zero or first-order absorption, a lag time (for HCQ only) and intercompartmental clearance between compartments. The between-subject variability (BSV) of the different pharmacokinetic parameters was estimated with an exponential error model while several error models were investigated to describe residual unexplained variability (additive, proportional, and mixed). The performance of the models was judged by both statistical and graphic methods [38]. The minimal value of the objective function was used to assess the goodness-of-fit. An increase in the goodness-of-fit is accompanied by a decrease in the objective function value, and this decrease is asymptotically distributed as a chi-square distribution. Furthermore, standard errors were calculated by use of the COVARIANCE option in NONMEM. For graphic model diagnostics, the following graphs were compared: observed concentrations (dependent variable [DV]) versus PRED, conditional weighted residuals (cWRES) versus time, cWRES versus PRED, individual predictions (IPRED) versus DV, and the normalized predictive distribution error (NPDE) versus PRED or time.

Thereafter a covariate selection using power model was performed with the following variables: age, body weight, height, body mass index (BMI), gender, AZT combination, and clinical unit (Intensive Care Unit or not). The diagnosis plots described above, the change in the objective function value and the change in parameter variability were used to select the covariates that improved the model. The likelihood ratio test was used to compare nested models with a significance level of *p* < 0.05 for forward addition of covariates and *p* < 0.001 for backward deletion of covariates. The final model estimated all parameters of the four compounds in one step. The trough blood concentrations at 48 h and 72 h were based on the final PK parameter estimates. A bootstrap (*n* = 500 datasets) was performed using Wings for NONMEM to construct confidence intervals by taking the lower 2.5% and the upper 97.5% value of each parameter. A visual predictive check (VPC) for each compound was performed by a simulation of 500 replicates, as implemented in the SIMULATION, SUBPROBLEM feature of NONMEM (Monte Carlo simulation).

Another set of simulations was performed to explore new dosing options. Simulations of 500 individuals based on the final model with between subject variability were conducted to describe the expected HCQ blood trough concentrations at 48 and 72 h following the first dose.

### 4.4. The Pharmacokinetic/Pharmacodynamic Analysis

The length of stay (duration of hospitalization), the number of deaths, and the clinical outcome were evaluated as pharmacodynamic parameters. The clinical outcome was a composite score where a poor clinical outcome was defined as one of the following: death, transfer to ICU, hospitalization lasting 10 days or more [39]. Other patients were considered as having a good clinical outcome. The relationships between the length of stay versus the BHCQ trough concentrations at 48 h, 72 h, or the HCQ total clearance were investigated with a linear regression model. The trough concentrations were analyzed with an analysis of variance with death or outcome as fixed factors. An empirical cumulative distribution function was computed for the length of stay according to different range of HCQ trough concentrations. An exploratory analysis was also performed with the PK parameters of the three metabolites. The statistical software was R version 4.0.3 [35].

## 5. Conclusions

In conclusion, our model-based simulations in COVID-19 patients with the final parameter estimates under a treatment with HCQ showed that at 48 h after the beginning of the treatment, BHCQ seemed to be related to clinical outcome and length of stay in hospital but not mortality in COVID-19 patients. A loading dose is required for treatment to be effective. A prospective clinical study should be now conducted in order to evaluate this effect coupled to therapeutic drug monitoring of HCQ.

## Figures and Tables

**Table 1:** Population characteristics.

		Overall	
		(*n* = 100)	
Gender			
	Male	66	−66.00%
	Female	34	−34.00%
Age (years)			
	Mean (SD)	60.7	−15.9
	Median [Min, Max]	62.5	[20.0, 94.0]
Height (m)			
	Mean (SD)	1.71	−0.094
	Median [Min, Max]	1.73	[1.52, 1.93]
Body weight (kg)			
	Mean (SD)	83.6	−20.1
	Median [Min, Max]	82	[37.5, 190]
Body Mass Index (kg/m^2^)			
	Mean (SD)	28.9	−5.41
	Median [Min, Max]	27.5	[18.5, 52.4]
Clinical Unit			
	Medicine	75	−75.00%
	ICU	25	−25.00%
Loading dose			
	Yes	42	−42.00%
	No	58	−58.00%
Azythromycin			
	No	22	−22.00%
	Yes	78	−78.00%
Length of stay (days)			
	Mean (SD)	15.8	−15.3
	Median [Min, Max]	11	[2.00, 88.0]
	Missing	35	−35.00%
Death			
	Yes	8	−8.00%
	No	92	−92
Clinical outcome			
	Poor	40	−40.00%
	Good	25	−25.00%
	Missing	35	−35.00%

**Table 2:** Population pharmacokinetic parameters of hydroxychloroquine and its metabolites.

PK Parameters	Unit	Value	RSE	Bootstrap	
			(%)	0.025	0.975
Lag (fixed)	h	0.389	-	-	-
KA (fixed)	1/h	1.15	-	-	-
CL/F HCQ	L/h	5.60	15.5	2.69	9.16
VP/F HCQ	L	1850	10.7	1560	2190
CL HCQ_DesCQ	L/h	4.99	17.5	3.73	7.57
CL DesCQ	L/h	49.8	23.7	30.8	85.8
CL HCQ_DesHCQ	L/h	9.63	10.3	8.29	11.5
CL DesHCQ	L/h	8.89	27.8	3.63	15.2
CL HCQ_DiDesCQ	L/h	1.84	10.8	1.61	2.17
CL DiDesCQ	L/h	11.6	27.2	6.21	20.5
**Inter Individual Variability (ω)**					
CL HCQ		1.327	9.2	0.899	1.790
VP HCQ		0.889	17.8	0.600	1.190
CL DesCQ		0.362	27	0.086	0.522
CL DesHCQ		0.860	23.1	0.316	1.290
CL DiDesCQ		0.953	18.1	0.621	1.300
**Residual Unexplained Variability (σ)**					
Proportional HCQ		0.448	9.6	0.355	0.543
Additive HCQ	μg/L	86.9	32.9	0.87	127
Proportional DesCQ		0.322	11.5	0.258	0.406
Additive DesCQ	μg/L	5.78	18	2.92	7.36
Proportional DesHCQ		0.428	12.7	0.345	0.542
Additive DesHCQ	μg/L	6.69	19.6	3.79	8.37
Proportional DiDescCQ		0.0574	13.7	0.046	0.072
Additive DiDescCQ	μg/L	2.49	16.7	0.62	3.21

HCQ: hydroxychloroquine; DesCQ: desethylchloroquine; DesHCQ: desethylhydroxychloroquine; DiDesCQ: di-desethylchloroquine; KA: first-order absorption rate constant; CL/F: apparent HCQ clearance; VP/F apparent volume of distribution of HCQ; CL HCQ_DesCQ: clearance from HCQ to DesCQ; CL DesCQ: clearance of DesCQ; CL HCQ_DesHCQ: clearance from HCQ to DesHCQ; CL DesHCQ: clearance of DesHCQ; CL HCQ_DiDesCQ: clearance from HCQ to DiDesCQ; CL DiDesCQ: clearance of DiDesCQ.

## Data Availability

The data are contained within the article and supplementary materials.

## Supplementary Materials

The following supporting information can be downloaded at: https://www.mdpi.com/article/10.3390/ph15020256/s1. Figure S1. Goodness-of-plots for the final HCQ, DesHCQ, DesCQ, DiDesCQ and population pharmacokinetic model.
