# Supplementary material for: Population Pharmacokinetics of Hydroxychloroquine and 3 Metabolites in COVID-19 Patients and Pharmacokinetic/Pharmacodynamic Application
Source: Pharmaceuticals (Basel). 2022 Feb 21;15(2):256. doi: 10.3390/ph15020256 (PMC8877570; doi:10.3390/ph15020256)
Supplement: Supplementary file 1 [file pharmaceuticals-15-00256-s001.zip › pharmaceuticals-1591411-supplementary.pdf]

Supplementary Materials

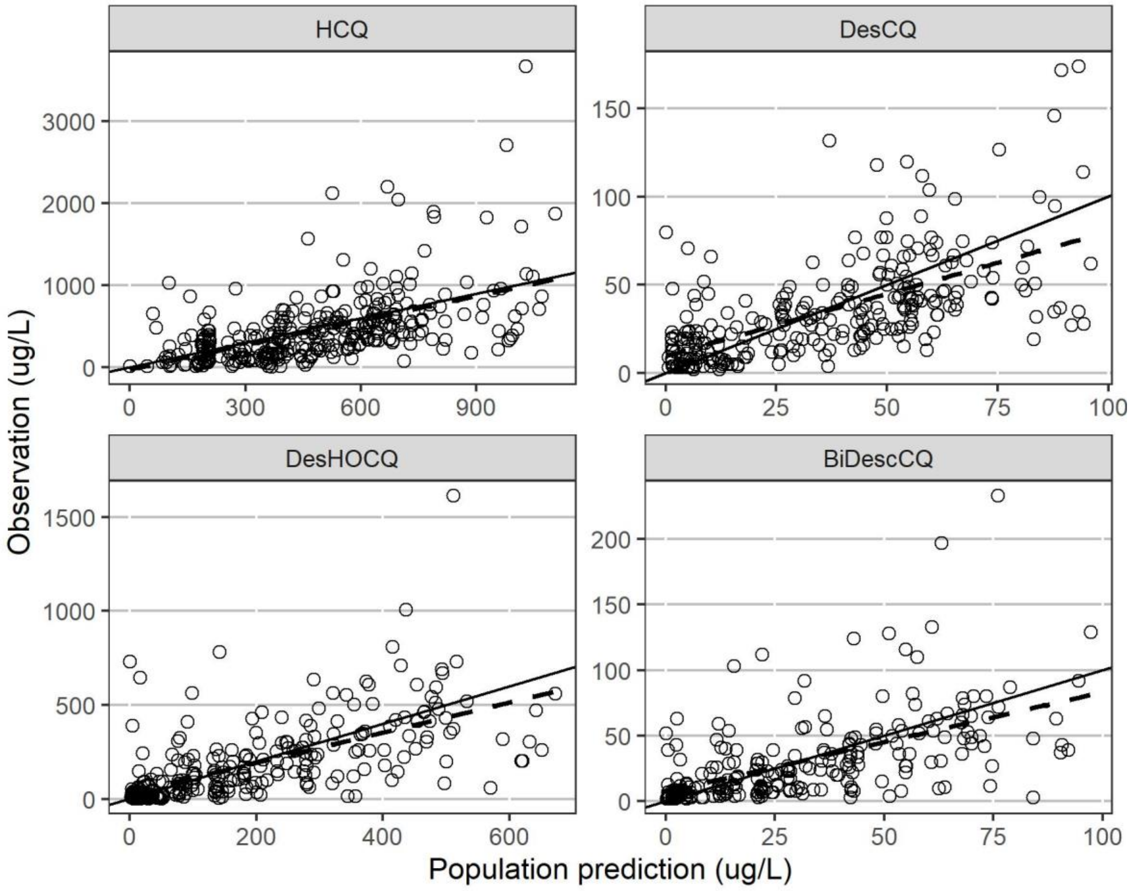

(A)

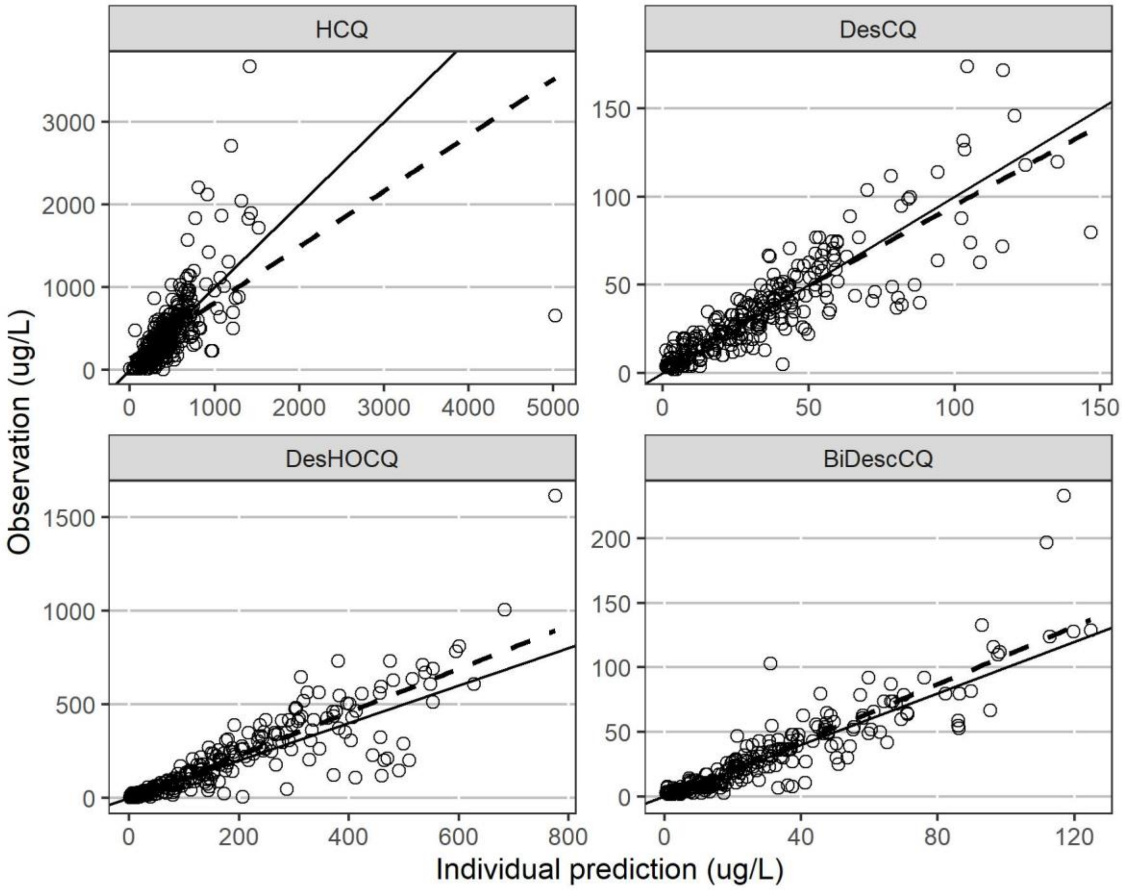

HCQ
DesCQ
DesHOCQ
BiDescCQ
Observation (ug/L)
Individual prediction (ug/L)

(B)

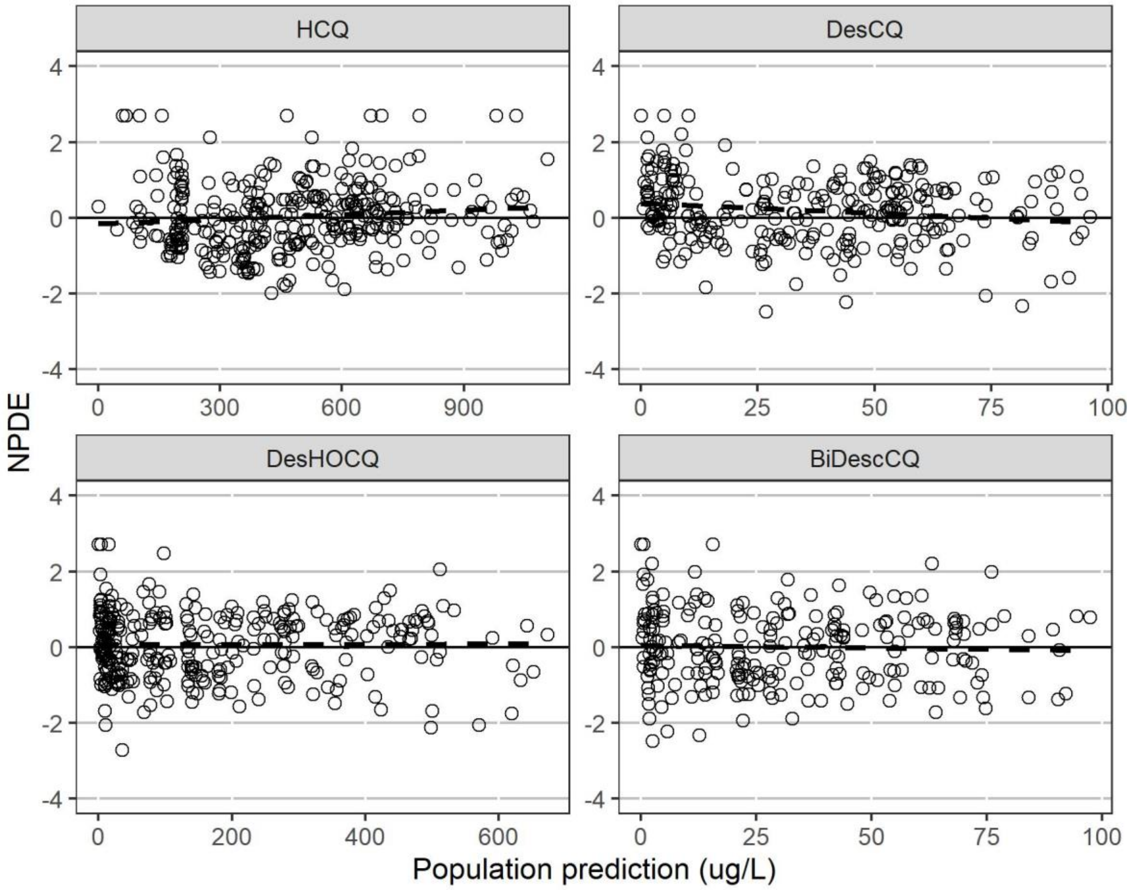

HCQ
DesCQ
DesHOCQ
BiDescCQ
NPDE
Population prediction (ug/L)

(C)

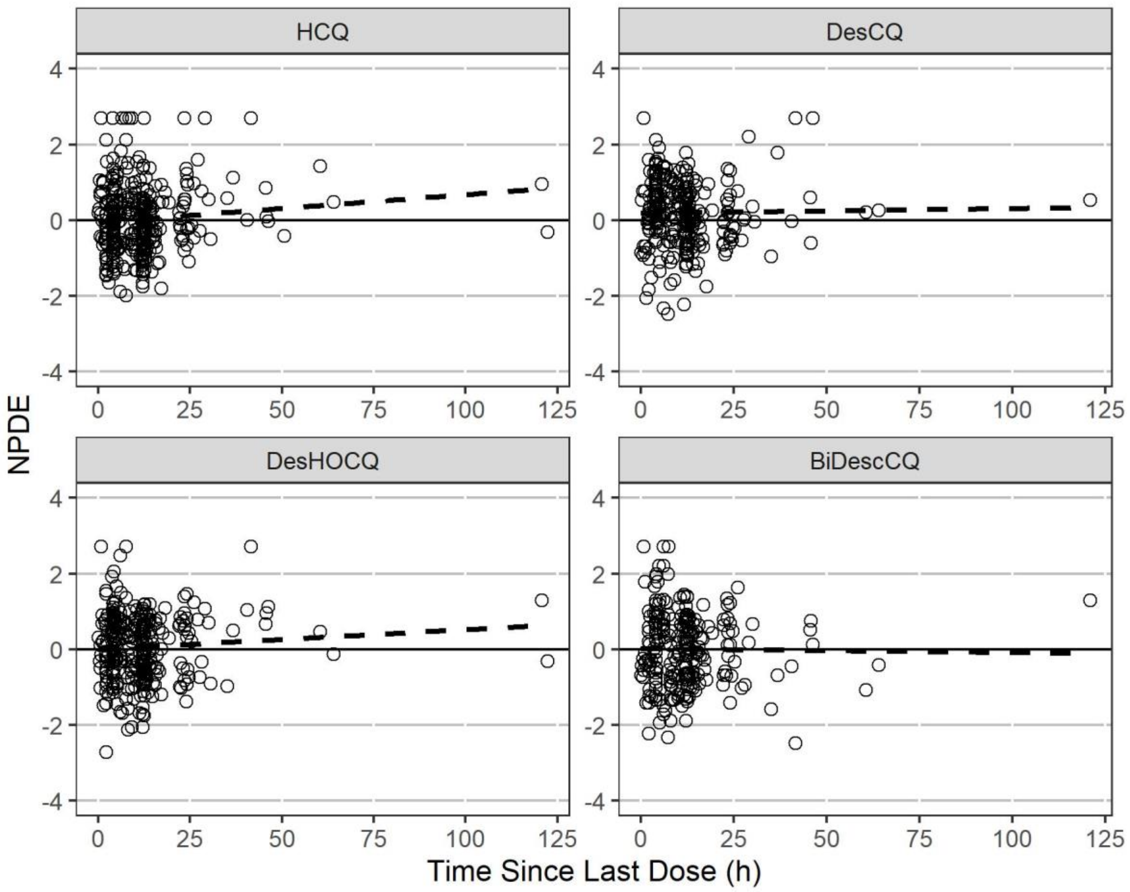

(D)

Figure S1. Goodness-of-plots for the final HCQ, DesHCQ, DesCQ, DiDesCQ and population pharmacokinetic model. (**A**) Observed concentrations (DV) versus population predictions (PRED), (**B**) Observed concentrations versus individual predictions (IPRED), (**C**) normalized predictive distribution error (NPDE) versus predictions, (**D**) NPDE vs. TIME. Under the figure: The line of identity (black line) and the linear regression (dotted line) are shown.
